# Modeling and Analysis of a Combined Stress-Vibration Fiber Bragg Grating Sensor

**DOI:** 10.3390/s18030743

**Published:** 2018-03-01

**Authors:** Kun Yao, Qijing Lin, Zhuangde Jiang, Na Zhao, Bian Tian, Peng Shi, Gang-Ding Peng

**Affiliations:** 1 State Key Laboratory of Mechanical Manufacturing Systems Engineering, Xi’an Jiaotong University, Xi’an 710049, China; vinsent@stu.xjtu.edu.cn (K.Y.); zdjiang@xjtu.edu.cn (Z.J.); zn2015@stu.xjtu.edu.cn (N.Z.); t.b12@mail.xjtu.edu.cn (B.T.); spxjy@mail.xjtu.edu.cn (P.S.); 2 Collaborative Innovation Center of High-End Manufacturing Equipment, Xi’an Jiaotong University, Xi’an 710054, China; 3 State Key Laboratory of Digital Manufacturing Equipment & Technology, Huazhong University of Science and Technology, Wuhan 430074, China; 4 State Key Laboratory of Fluid Power and Mechatronic Systems, Zhejiang University, Hangzhou 310027, China; 5 School of Electrical Engineering and Telecommunications, UNSW, Sydney, NSW 2052, Australia; g.peng@unsw.edu.au

**Keywords:** FBG sensor, vibration sensing, stress sensing, analytical theory, FEM

## Abstract

A combined stress-vibration sensor was developed to measure stress and vibration simultaneously based on fiber Bragg grating (FBG) technology. The sensor is composed of two FBGs and a stainless steel plate with a special design. The two FBGs sense vibration and stress and the sensor can realize temperature compensation by itself. The stainless steel plate can significantly increase sensitivity of vibration measurement. Theoretical analysis and Finite Element Method (FEM) were used to analyze the sensor’s working mechanism. As demonstrated with analysis, the obtained sensor has working range of 0–6000 Hz for vibration sensing and 0–100 MPa for stress sensing, respectively. The corresponding sensitivity for vibration is 0.46 pm/g and the resulted stress sensitivity is 5.94 pm/MPa, while the nonlinearity error for vibration and stress measurement is 0.77% and 1.02%, respectively. Compared to general FBGs, the vibration sensitivity of this sensor is 26.2 times higher. Therefore, the developed sensor can be used to concurrently detect vibration and stress. As this sensor has height of 1 mm and weight of 1.15 g, it is beneficial for minimization and integration.

## 1. Introduction

Vibration and stress are two important parameters in mechanical condition monitoring and fault detection, which have been successfully applied in various machinery industries to improve operation reliability including aircraft engines, power station turbines and industrial robots [1,2,3]. Compared with traditional electronic sensors, optical fiber sensors have advantages such as insensitivity to electromagnetic interference, resistance to harsh environments, high measurement accuracy with high signal to noise ratio, availability for multi-parameter sensing, more flexible sensor distribution for remote monitoring and capability for distributed sensing [4,5,6,7,8,9].

The fiber Bragg grating (FBG) sensors are small in size, corrosion-resistant and easy to be multiplexed [10]. They have been widely used in vibration or stress detection. Au et al. [11] proposed a vibration sensor based on tapered plate FBG. The sensitivity for measuring frequency below 150 Hz is 18.93 με/g. The weight of the sensor is more than 29.8 g and its height is more than 32 mm. Khan et al. [12] presented a sensor based on an L-shaped FBG, whose sensitivity is 46 pm/g with resonant frequency up to 50 Hz. Its sensitivity increases to 306 pm/g while the frequency is larger than 150 Hz. The weight of the sensor is 23 g. Weng et al. [13] presented a compact FBG vibration sensor for oil or gas exploration whose length is larger than 30 mm. Stefani et al. [14,15] fabricated FBGs for 1550 nm and 850 nm operations based on polymer and presented an accelerometer whose resonance frequency is up to 3 kHz with a sensitivity of 19 pm/g. Xu et al. [16] presented a bundle-structure riser stress sensor based on FBG, whose measure range is 0–132.7 MPa. Pospori et al. [17] investigated the thermal annealing effects on the stress and strain sensitivities of polymer FBG sensors. Optical Fiber sensors can also be used for sensing stress/strain inside of the fibrous composite materials [18,19,20,21].

Most of these sensors can only sense vibration or stress. However, the vibration and stress often coexist at one measurement point, and these two signals interfere with each other, making it difficult to measure both of them simultaneously. Many sensors have large size or weight, making it difficult to be integrated and lightweight. In addition, it may have a wide range of temperature change while sensing vibration or stress under high temperature. This will increase the sensing error or even make the sensor unusable. Therefore it is necessary to realize temperature compensation when the sensor is used in the condition of high temerature. 

The use of the combined stress-vibration sensor can help to reduce the needed number of sensors and simplify the wiring layout. If the height of a sensor is small and the weight is light, it is helpful to be integrated, miniaturized and light weighted. Take the aircraft engines as an example, sensors are needed to monitor health to extend their service time. However, the engines need to provide additional space for sensors installation. This will increase their size and potential danger reducing their usability. The use of the combined stress-vibration sensor can help to reduce the needed additional space for installation. In this work, a combined stress-vibration sensor is developed to measure vibration and stress simultaneously based on FBG technology. The sensor is composed of two FBGs and a specially designed stainless steel plate, which can significantly increase the sensitivity for vibration sensing. Theoretical analysis and FEM were carried out to analyze the sensor’s sensing properties. The sensor can achieve temperature compensation by itself through reasonable structural design.

## 2. The Designed Sensor

### 2.1. Sensor Design

**Figure 1:**
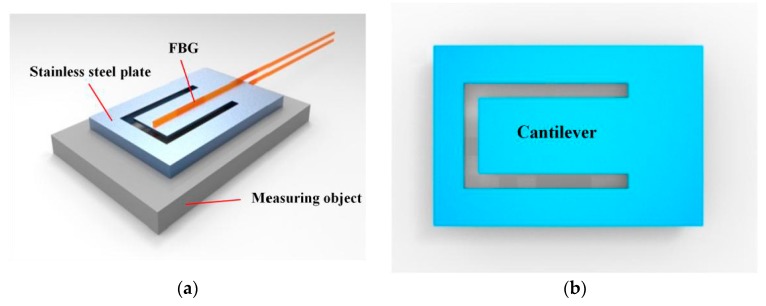
The designed sensor. (**a**) Components of the sensor; (**b**) The designed stainless steel plate.

The structure of the designed sensor is shown in Figure 1a. The combined stress-vibration sensor is proposed to sense stress and vibration simultaneously. The sensor is composed of two fiber gratings in different fibers and a specially designed stainless steel plate with a rectangular cantilever, as shown in Figure 1b.

**Figure 2:**
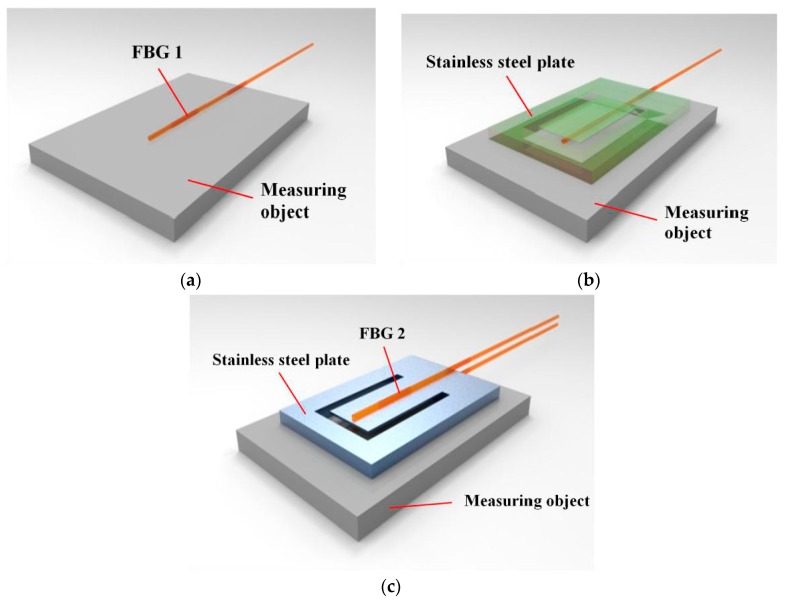
The pasting steps of the designed sensor. (**a**) Pasting of the FBG 1; (**b**) Pasting of the stainless steel plate; (**c**) Pasting of the FBG 2.

The pasting steps of the designed sensor are shown in Figure 2. First, one of the fiber gratings, FBG 1, is pasted on the surface of the measuring object (shown in Figure 2a). Then the plate is pasted on the measuring object surface (shown in Figure 2b). Finally, FBG 2 is pasted on the top surface of the cantilever (shown in Figure 2c). FBG 1 is used to sense stress of the measuring object and FBG 2 is used to sense vibration. The FBG 2 also acts as a temperature compensator, which helps to compensate the error of stress sensing caused by the change of measuring environment temperature.

Considering the resolution and fabrication technology, the length, width and thickness of the plate are designed as 18 mm, 12 mm and 1 mm, respectively. The geometry parameters of the cantilever are 10 mm, 5 mm and 0.2 mm, respectively. The lengths of the two FBGs are both 4 mm. The details of size designing will be explained in Section 3.1. According to the sensor’s size and density, the total weight of the sensor is about 1.15 g. So the designed sensor is very light and small.

However, it is difficult to calculate the output of the FBGs directly based on current mechanics theory because of the complicated structure. Therefore, a new calculation model for the sensor is needed.

### 2.2. Theoretical Analysis of FBG

The loaded strain can be obtained by observing the change of the center wavelength of the FBG. The FBG’s center wavelength λ is represented as:

(1) $$\lambda =2n_{eff}\Lambda$$

where $n_{eff}$ is the effective refractive index of the FBG; Λ is the grating period. FBGs are sensitive to its axial strain. Strain of the other directions can hardly affect FBGs’ output [22,23]. While the axial strain changes, Λ of the FBG changes accordingly, and its refractive index also changes because of the photoelastic effect. Equation (2) shows how λ changes with the axial strain:

(2) $$\{\begin{array}{l} \Delta \lambda =\lambda (1+\gamma )\varepsilon \\ \gamma =-\frac{n_{eff}{}^{2}}{2}[(1-\nu )p_{12}-\nu p_{11}] \end{array}$$

where γ is the effective photoelastic coefficient; ν is the Poisson’s ratio; $p_{12}$ and $p_{11}$ are the elasto-optical coefficients. As for the silica fiber used in this work, γ is −0.22, and Δλ/ε equals to 1.2 pm/(με) when *λ* *≈* 1550 nm.

Because of the thermo-optic effect of refractive index, the center wavelength changes with the sensing environment temperature as well. As for the silica fiber used in this work, the temperature coefficient is about 10 pm/K when *λ* *≈* 1550 nm.

### 2.3. Theoretical Analysis of Cantilever with Vibration

#### 2.3.1. Theoretical Model

**Figure 3:**
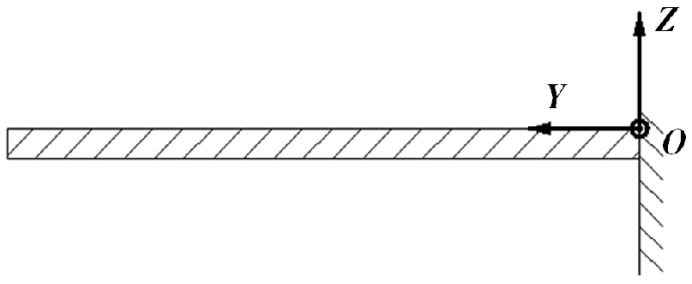
The schematic diagram of Bernoulli-Euler Cantilever.

Because thickness of the cantilever is much smaller than its length, bending will be the main deformation. The influence of shear deformation and the rotation inertia of the interface are ignored. The cantilever is simplified as a Bernoulli-Euler Cantilever, as shown in Figure 3.

According to Thomson [24], if there is no initial load or initial displacement, the response of Bernoulli-Euler Cantilever to arbitrary load can be presented as:

(3) $$z(y,t)=\sum_{j=1}^{\infty }\frac{1}{\omega_{j}}Y_{j}(y)\int_{0}^{l}Y_{j}(y)\times \int_{0}^{t}[p(y,\tau )-\frac{\partial }{\partial y}m(y,\tau )]\sin\omega_{j}(t-\tau )d\tau dy$$

where z(y,t) is the lateral displacement of the cantilever at coordinate of *y* and time of *t*; $\omega_{j}$ is the natural frequency of *j* order; l is the length of the cantilever; p(y,τ) is the external force per unit length, and it is parallel to *Z*-axis; m(y,τ) is the external torque per unit length; $Y_{j}(y)$ is the main vibration type of *j* order can be presented as:

(4) $$\{\begin{array}{l} Y_{j}(y)=C_{1,j}\cos\beta_{j}y+C_{2,j}\sin\beta_{j}y+C_{3,j}\cosh\beta_{j}y+C_{4,j}\sinh\beta_{j}y\\ \beta_{j}{}^{4}=\frac{\rho hb\omega_{j}{}^{2}}{EJ} \end{array}$$

where $C_{1,j}$, $C_{2,j}$, $C_{3,j}$ and $C_{4,j}$ are constants; ρ is the density of the cantilever; *h* is the thickness of the cantilever; *b* is the width; *E* is the Young’s Modulus of the cantilever; J is the area moment of inertia. $C_{1,j}$, $C_{2,j}$, $C_{3,j}$, $C_{4,j}$ and $\omega_{j}$ are determined by the boundary conditions and the normalization of main vibration type.

According to the mechanics of materials, the bending moment M(y,t), the stress σ(y,t) and the strain ε(y,t) of the cantilever’s top surface can be represented as:

(5) $$\{\begin{array}{l} M(y,t)=EJ\frac{\partial^{2}z(y,t)}{\partial y^{2}}\\ \sigma (y,t)=\frac{M(y,t)}{J}\times \frac{1}{2}h\\ \varepsilon (y,t)=\frac{\sigma }{E} \end{array}$$

The strain of cantilever’s top surface changes with the vibration. According to Equations (3) and (5), ε(y,t) can be represented as:

(6) $$\varepsilon (y,t)=\frac{h}{2}\frac{\partial^{2}}{\partial y^{2}}(\sum_{j=1}^{\infty }\frac{1}{\omega_{j}}Y_{j}(y)\int_{0}^{l}Y_{j}(y)\times \int_{0}^{t}[p(y,\tau )-\frac{\partial }{\partial y}m(y,\tau )]\sin\omega_{j}(t-\tau )d\tau dy)$$

The FBG 2 is pasted on the top surface of the cantilever as shown in Figure 1c. Because the Young’s Modulus of the stainless steel plate is much larger than that of the FBG, the pasted FBG 2 can hardly affect the strain distribution of the cantilever. In other words, the strain of FBG 2 equals to the strain of the cantilever pasted point. So sensing vibration can be simplified as sensing the strain of cantilever’s top surface.

#### 2.3.2. Theoretical Analysis

Based on the initial boundary conditions of the cantilever, the solution of Equation (4), $\omega_{i}$ and $Y_{i}(y)$ can be obtained,

(7) $$\{\begin{array}{ll} \beta_{1}l=1.875 & \\ \beta_{2}l=4.694 & \\ \beta_{i}l\approx (i-\frac{1}{2})\pi & i=3,4,\ldots \\ \omega_{i}={\left(\beta_{i}l\right)}^{2}\sqrt{\frac{EJ}{\rho bhl^{4}}} & i=1,2,\ldots \\ Y_{i}(y){=C}_{j}[\cos\beta_{i}y-\cosh\beta_{i}+r_{i}(\sin\beta_{i}y-\sinh\beta_{i}y)] & i=1,2,\ldots \end{array}$$

where $r_{i}=-\frac{\cos\beta_{i}l+\cosh\beta_{i}l}{\sin\beta_{i}l+\sinh\beta_{i}l}$; $C_{j}$ is a constant.

In this work, p(y,τ) is inertia force produced by vibration. m(y,τ) equals to zero all the time. If p(y,t) is described as an sinusoidal function of time, $p(y,t)=A_{p}\cdot \sin(\omega_{p}t)$, Equation (3) can be simplified as:

(8) $$\{\begin{array}{l} z(y,t)=A_{z}(y)\times (\omega_{j}\sin\omega_{p}t-\omega_{p}\sin\omega_{j}t)\\ A_{z}(y)=\sum_{j=1}^{\infty }\frac{2r_{j}C_{j}A_{p}Y_{j}(y)}{\beta_{j}\omega_{j}(\omega_{j}{}^{2}-\omega_{p}{}^{2})} \end{array}$$

If $\omega_{p}$ is much smaller than $\omega_{j}$, z(y,t) can be simplified as a sinusoidal function. Its radian frequency and phase equal to that of p(y,t). If $p(y,t)=A_{p,1}\sin(\omega_{p,1}t)+A_{p,2}\sin(\omega_{p,2}t)+\ldots$, z(y,t) will equal to the linearly superposed of results which are obtained by inputting $A_{p,1}\sin(\omega_{p,1}t)$, $A_{p,2}\sin(\omega_{p,2}t)$, $A_{p,3}\sin(\omega_{p,3}t)$, … respectively.

Based on Equations (6)–(8), the relationship between ε(y,t) and p(y,t) is obtained:

(9) $$\{\begin{array}{l} \varepsilon (y,t)=A_{\varepsilon }(y)\times (\omega_{j}\sin\omega_{p}t-\omega_{p}\sin\omega_{j}t)\\ A_{\varepsilon }(y)=\sum_{j=1}^{\infty }-\frac{hr_{j}C_{j}{}^{2}\beta_{j}A_{p}[\cos\beta_{j}y+\cosh\beta_{j}y+r_{j}(\sin\beta_{j}y+\sinh\beta_{j}y)]}{\omega_{j}(\omega_{j}{}^{2}-\omega_{p}{}^{2})} \end{array}$$

According to the normalization condition $\int_{0}^{l}\rho hbY_{j}{}^{2}dy=1$, where j=1,2,3,…, $C_{j}$ can be confirmed. 

#### 2.3.3. Finite Element Model

**Figure 4:**
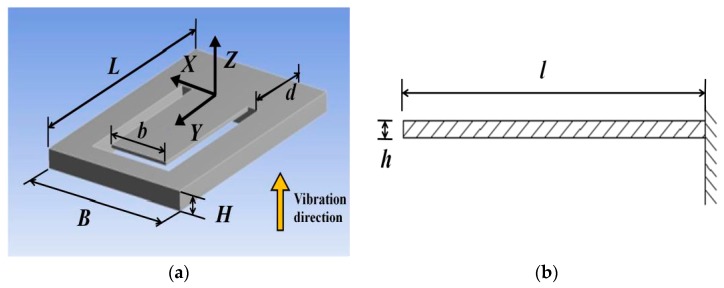
Geometry parameters of the stainless steel plate. (**a**) Stereoscopic view of the stainless steel plate. (**b**) Sectional view of the cantilever.

We used ANSYS Workbench 15.0 to analyze the distribution of cantilever strain and stress. The built model is shown in Figure 4. Its parameters are shown in Table 1. The bottom of the model is fixed. The set vibration is parallel to the *Z*-axis. Use the “Resonance Analysis” module to analyze the cantilever’s responses to harmonic excitations.

### 2.4. Theoretical Analysis of Measuring Object with Stress

#### 2.4.1. Theoretical Model

**Figure 5:**
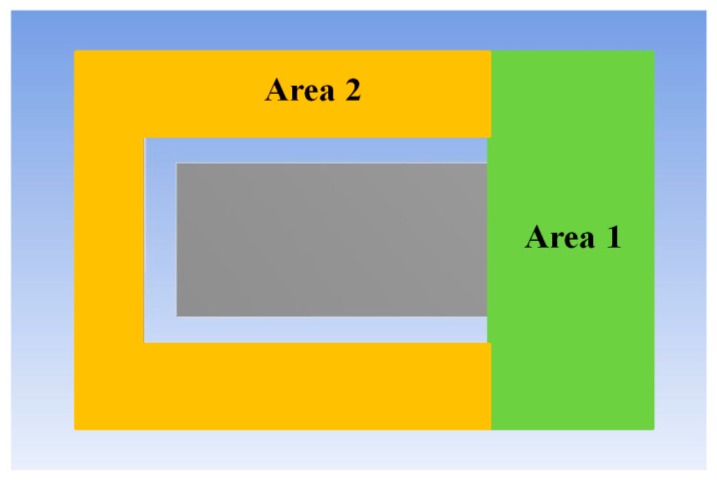
The pasted area of the stainless steel plate for sensing stress.

As shown in Figure 2, the sensor is pasted on the surface of the measuring object. The pasted sensor will affect the measuring object’s surface stress distribution. To reduce the impact, only area 1 of the bottom of the plate is pasted on the surface of the measuring object, as shown in Figure 5. There’s no constraint between area 2 and the surface of the measuring object.

The FBG 1 is pasted on the surface of the measuring object, as shown in Figure 2a. It has the same strain as the pasted point of the measuring object. Thus, the FBG 1 can be used to sense the strain of the measuring object’s surface.

When the environment temperature changes, the center wavelength of the FBG 1 and FBG 2 will drift according to Section 2.2. In general, the change rate of the temperature is much smaller than that of the strain caused by vibration. If the signal sensed by FBG 2 is analyzed with the short-time Fourier transform, the value with the frequency to be zero represents temperature and the other values represent the vibration. Remove the temperature value from the signal sensed by FBG 1, the signal of the measuring object’s surface strain can be obtained.

Because the FBG 1 and FBG 2 are close to each other it can be considered that they are under the same measuring environment temperature. Therefore, the measured temperature by FBG 2 can be used for the temperature compensation of the FBG 1.

#### 2.4.2. Finite Element Model

**Figure 6:**
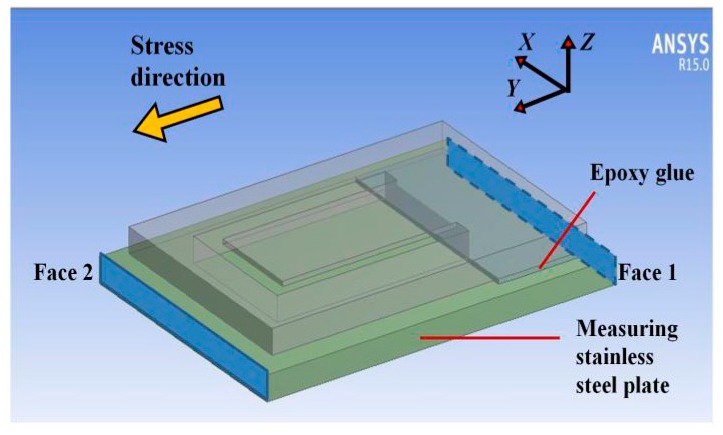
Finite element model and boundary conditions of the designed sensor and the measuring object for stress sensing.

Use ANSYS Workbench 15.0 analyze the distribution of strain and stress of the sensor. As shown in Figure 6, the built model contains the designed stainless steel plate, 0.1 mm thick epoxy glue and 1 mm thick measuring object. The material of the measuring object is set as stainless steel. All the contacts are set as bonded. The face 1 of the stainless steel plate is ‘displacement’ fixed. The face 2 is loaded with 100 Mpa pressure. Use the “Static Structural” module to analyze the strain and stress. 

## 3. Results and Discussion

### 3.1. Theoretical Analysis and FEM for Vibration Sensing

**Figure 7:**
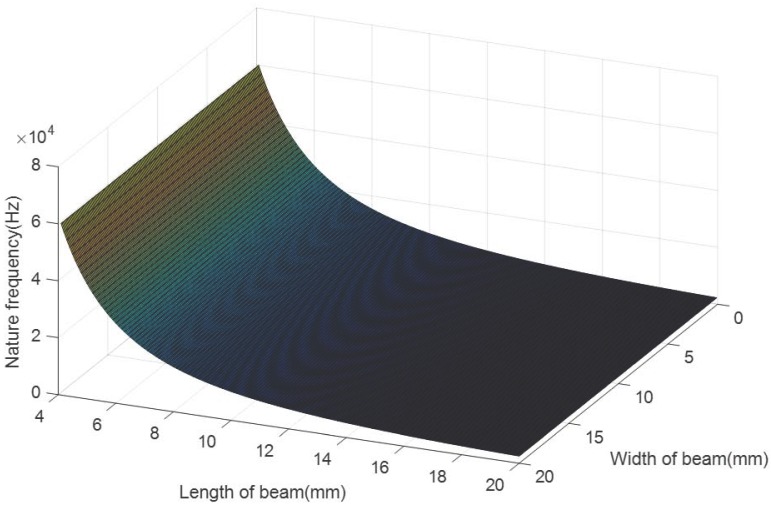
The cantilever’s first natural frequency changes with its length and width.

For the convenience of fabrication, the cantilever of the sensor is designed in a rectangular shape. To ensure that the sensor is miniaturized and lightweight, the thickness of the steel plate is designed as 1 mm and the thickness of the cantilever is 0.2 mm. The lengths of FBGs are 4 mm. Figure 7 shows the cantilever’s first natural frequency changing with its length and width. It can be seen that the cantilever’s first natural frequency increases with its length. The width has less effect on the first natural frequency. The designed sensor is expected to be able to sense vibration in high frequency, so the cantilever is requested to have high natural frequency to ensure that the measurement linearity error is acceptable. According to Figure 7, its first natural frequency is about 10,000 Hz when the length is 10 mm.

**Figure 8:**
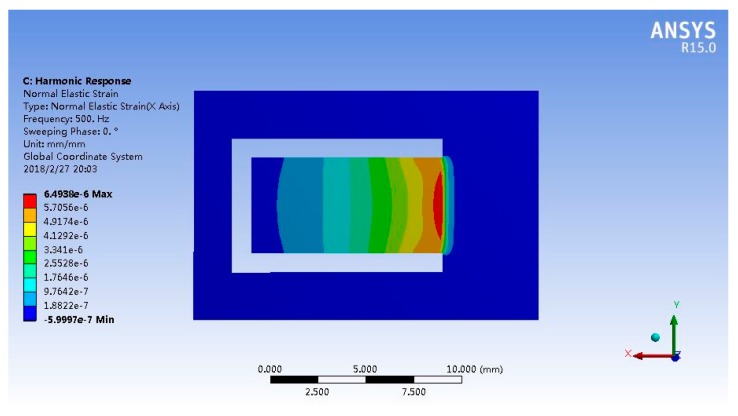
The strain distribution of the designed stainless steel plate.

**Figure 9:**
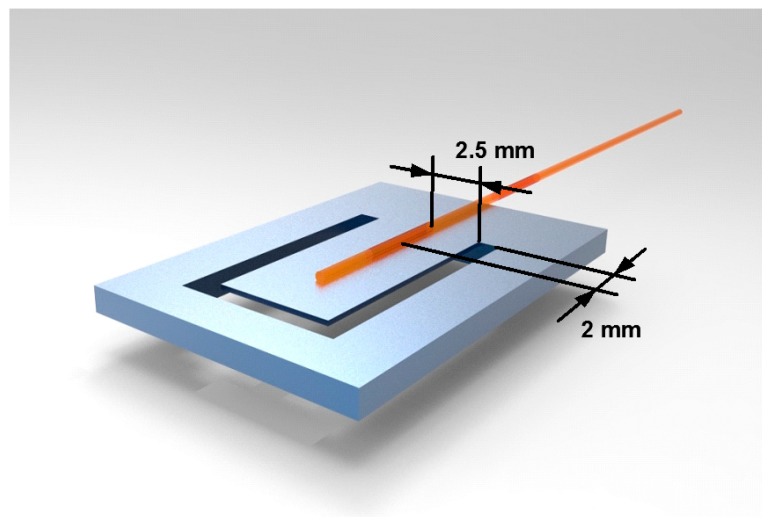
The paste of FBG 2 on the cantilever and the center coordinates of FBG 2.

The strain of FBG 2 is assumed to be uniform and equivalent to its center strain [25] while doing theoretical analysis and FEM. The maximum strain occurs at the root of the cantilever, as shown in Figure 8. To achieve a high-sensitivity sensor, the FBG 2 is pasted at the root of the cantilever. Its center is 2 mm away from the fixed end and 2.5 mm away from the side of the cantilever, as shown in Figure 9. However, because the strain of the cantilever’s top surface is different, the strain of FBG 2 is not uniform actually. This will cause sensing error. Because the strain of FBG 2 is continuous and smooth along its axis, its reflectance spectrum will be wider and will not split significantly into multiple reflection peaks. For example, according to theoretical analysis and FEM, the maximum and minimum strains of FBG 2 are 59.1 με and 21.7 με when the frequency and acceleration of vibration are 500 Hz and 100 g, respectively. The boundaries of FBG’s reflectance peak has changed by 70.9 pm and 26 pm respectively. Its center has changed by 48.5 pm. According to the center strain (38.8 με) of FBG 2, the reflectance peak has changed by 46.6 pm. The error of FBG 2 reflectance peak shift is 4% and increases slightly as the vibration acceleration decreasing.

**Figure 10:**
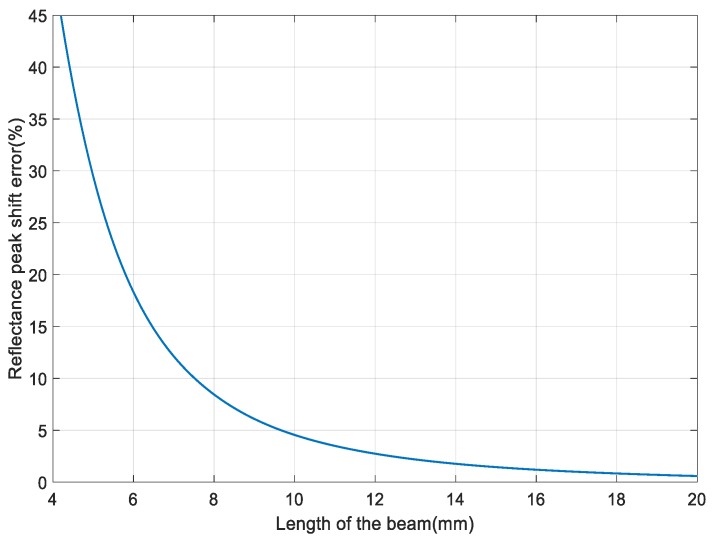
The reflectance peak shift error changes with the length of the cantilever.

Figure 10 shows the error of reflectance peak shift changing with the length of the cantilever. The vibration frequency is set as 500 Hz and the vibration acceleration is set as 100 g. It can be seen that the error increases significantly as the length decreasing when it is less than 10 mm. To ensure that the designed sensor has good sensitivity, the length of the cantilever need not be less than 10 mm.

Overall, considering the sensing of high-frequency vibration and the reflectance peak shift error, the optimal length of the cantilever is selected as 10 mm. Its width is designed to be half the length (5 mm) to prevent the cantilever from torsional vibrations. With the designed size, the strain of FBG 2 can be assumed to be uniform and equal to its center strain.

Based on Equations (3), (4), (7) and (8), the amplitude of the tip of the cantilever can be obtained. When the vibration acceleration is 100 g and the frequency is 6000 Hz, the amplitude is 0.042 mm, which is much smaller than the thickness of the stainless steel plate. Thus the tip of the beam will not hit the measuring object.

**Figure 11:**
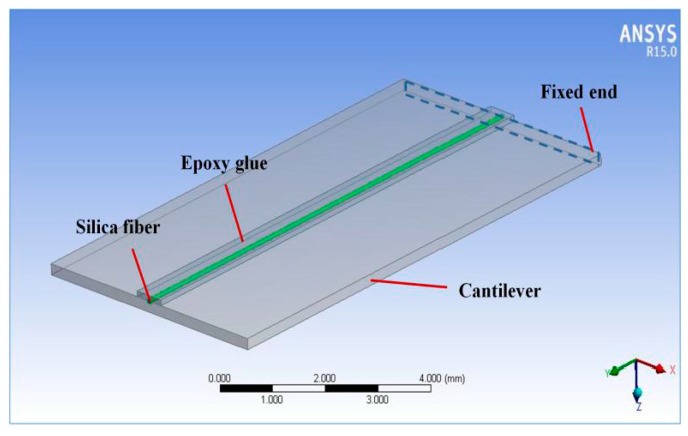
The model of cantilever with a silica fiber pasted on its surface.

**Figure 12:**
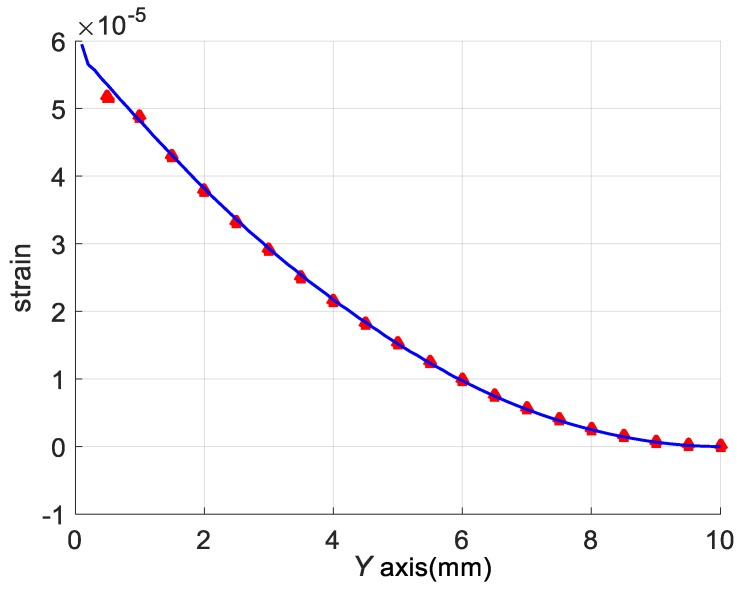
The strain of the pasted fiber and the cantilever’s top surface.

The strain of FBG 2 is assumed to equal to the strain of the cantilever’s top surface in theoretical analysis. However, they are not exactly the same in experiments. This will cause error when the sensor senses vibration. Use ANSYS Workbench 15.0 to build a model which has a silica fiber pasted on the cantilever’s top surface, as shown in Figure 11. The silica fiber is embedded in the cantilever surface and covered by epoxy glue. The vibration frequency and acceleration are set as 500 Hz and 100 g respectively. The results of this simulation are shown in Figure 12. The strain of the fiber is shown as red points. The blue curve represents the cantilever surface strain without the fiber. 

To show the strain intuitively, the coordinate origin is set at the fixed end of the cantilever, as shown in Figure 4a. According to Figure 9, the FBG 2 is pasted on the cantilever from 0 to 4 mm. The coordinates of the FBG 2 are *x* = 0 mm, *y* = 2 mm. The maximum equivalent strain error of FBG 2 obtained from Figure 12 is 3.5%. So the red points fit the blue curve very well, and it is suitable to represent the strain of FBG 2 by the strain of cantilever surface to simplify the theoretical analysis and FEM.

**Figure 13:**
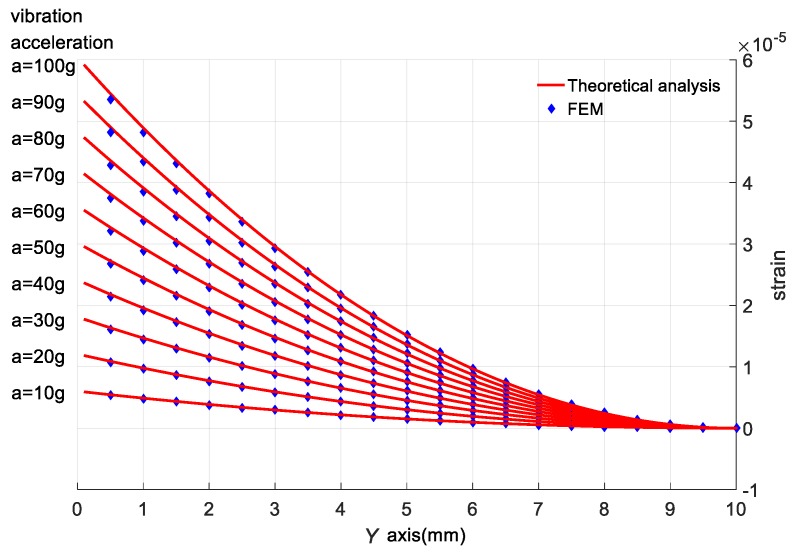
The strain of the cantilever top surface obtained by theoretical analysis and FEM with different vibration acceleration.

Figure 13 shows the strain of the cantilever’s top surface along the *Y*-axis. The strain is obtained respectively by theoretical analysis and FEM with different vibration accelerations. The vibration frequency is set as 500 Hz. It can be seen that the strain decreases with the *y*-coordinate increasing. The larger the vibration acceleration is, the lager the stress will be.

**Figure 14:**
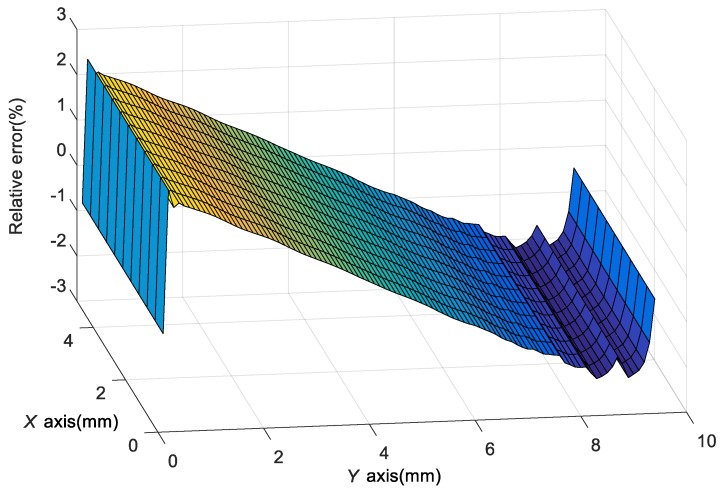
The relative error of the strain obtained by theoretical analysis and FEM.

Figure 14 shows the relative error of the cantilever’s top surface strain obtained respectively by theoretical analysis and FEM with the vibration frequency and acceleration set as 500 Hz and 100 g. It can be seen that the relative error is less than 2.5% and it decreases with the y-coordinate increasing. However, it hardly changes with the *x*-coordinate. The error comes from the simplification of the theoretical model. The cantilever is simplified as a Bernoulli-Euler Cantilever and only displacement in *Z*-axis direction is considered. However, displacement in *X*-axis and *Y*-axis directions is also considered in FEM. So the result of the theoretical analysis cannot fit the result of the FEM completely.

**Figure 15:**
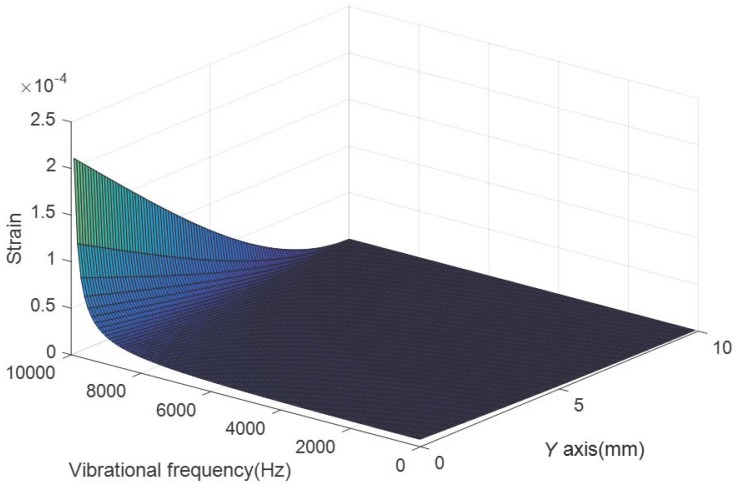
The relationship between the vibration frequency and the strain of the cantilever’s top surface.

Figure 15 shows the relationship between the cantilever’s top surface strain and the vibration frequency. The vibration acceleration is set as 10 g. The first natural frequency of the cantilever is 10,129 Hz according to the result of theoretical analysis. It can be seen from Figure 15 that the strain have good linearity between 0 and 6000 Hz and it increases significantly when the frequency approaches to 10,129 Hz. Thus the vibration sensing range of the designed sensor is set as 0–6000 Hz. 

The above analysis is in the steady stage of the cantilever’s steady response. In this stage, the vibration frequency of the cantilever equals to the frequency of excitation [24]. Before the steady stage, the cantilever will experience the transitional stage. In this stage, the sensor senses the vibration of the measuring object and the free vibration of the cantilever. Length of this stage depends on the natural frequency of the cantilever and the system’s damping which can be obtained in experiments. Overall, the designed sensor is more suitable to measuring steady-state vibration.

Because of the cantilever’s own dynamics, it will continue to vibrate in a short time after the measuring object stops vibrating. In this stage, the vibration of the cantilever is free with initial energy. It is equivalent to the free vibration caused by an impulse input and the higher its natural frequency is, the shorter this duration will be. The designed sensor has a high natural frequency, so the vibration of the cantilever will decay quickly.

### 3.2. The Sensor Performance for Vibration Sensing

#### 3.2.1. Measurement Linearity of the Sensor

According to Section 2.2, $\frac{\Delta \lambda }{\varepsilon }$ equals to 1.2 pm/(με) when the center wavelength of FBG is around 1550 nm. Based on the Equation (9), the relationship between the change of the center wavelength, Δλ, and p(y,t) can be obtained. The strain of the cantilever’s surface slightly increases as the vibration frequency increasing (0–6000 Hz). To reduce this linearity error, the least squares method is used. A third order function is used to fit the relationship between Δλ and p(y,t). The third order function is represented as:

(10) $$\Delta \lambda =1.0852\times 10^{-13}af^{3}-9.7025\times 10^{-11}af^{2}+8.7818\times 10^{-7}af+0.459729a$$

What needs to be clear is that Equation (10) is a curve fitted equation. Δλ, a and f in Equation (10) only represent the values of wavelength changes, vibration acceleration and vibration frequency. They are dimensionless.

**Figure 16:**
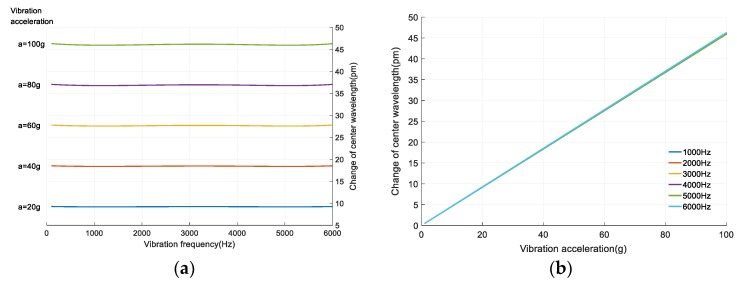
The changes of the center wavelength with the increase of vibration frequency and acceleration. (**a**) The f − Δλ curve. (**b**) The a − Δλ curve.

According to Equations (9) and (10) and Section 2.2, a certain Δλ(f = 100 Hz) is used as the reference to reduce the linear errors. The better linearity f − Δλ curve and a − Δλ curve are obtained, as shown in Figure 16. It shows that Δλ hardly changes with f increasing but increases linearly with the increase of a. Its maximum linearity error is 0.77%.

#### 3.2.2. Sensing Sensitivity

**Figure 17:**
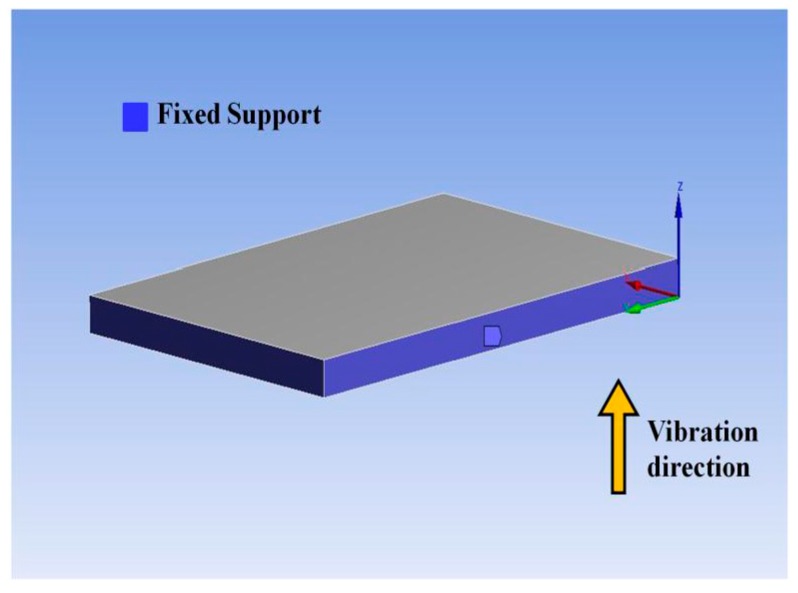
The normal stainless steel and its boundary conditions.

**Figure 18:**
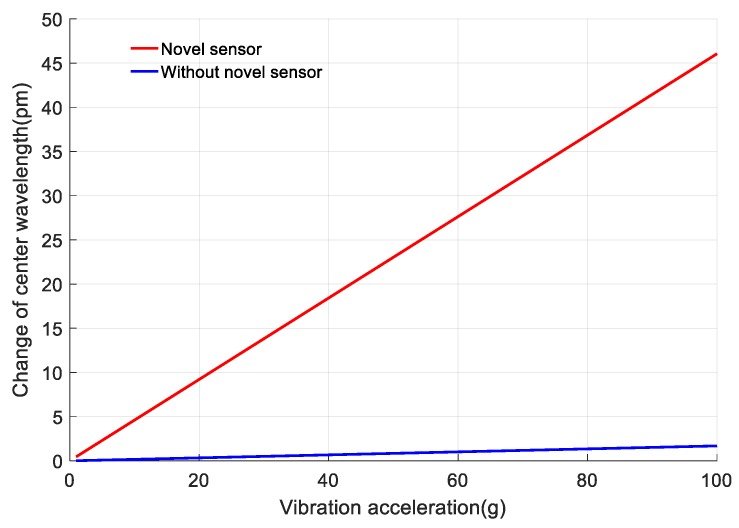
The comparison of sensing sensitivity of designed sensor and a normal FBG.

One of the important roles of the sensor is to increase the sensitivity for vibration sensing. *Δλ* of FBGs without the cantilever is much smaller. Take a FBG which is posted on the normal stainless steel for example. The steel’s length, width and height are set as 18 mm, 12 mm and 1 mm respectively. Its four sides are fixed. The loaded vibration parallel to *Z*-axis, as shown in Figure 17. The vibration frequency is set as 500 Hz. The FBG is pasted on the maximum-strain point of the steel, and the change of *Δλ* with the increase of the vibration acceleration is shown as the blue curve in Figure 18. The red curve stands for *Δλ* of the designed sensor. The designed sensor’s sensitivity is 0.46 pm/g, which is 26.2 times higher than that of the FBG pasted on the normal steel. Overall, the designed sensor significantly increases the sensitivity for vibration sensing with small size (1 mm thick) and light weight (1.15 g).

**Figure 19:**
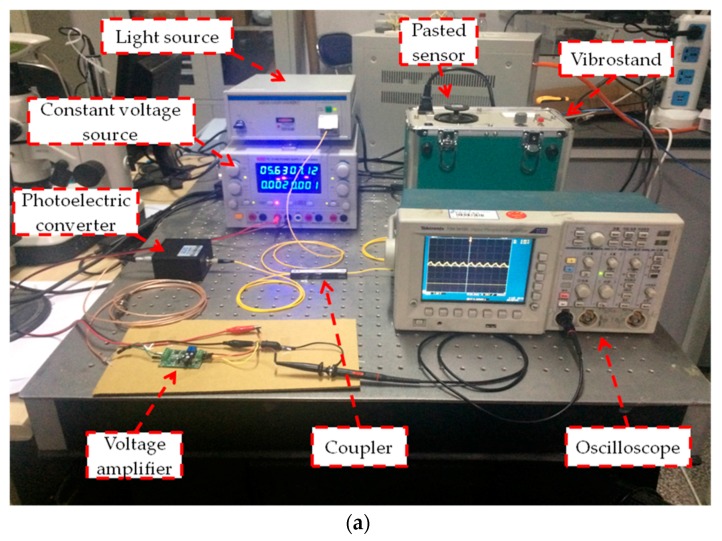

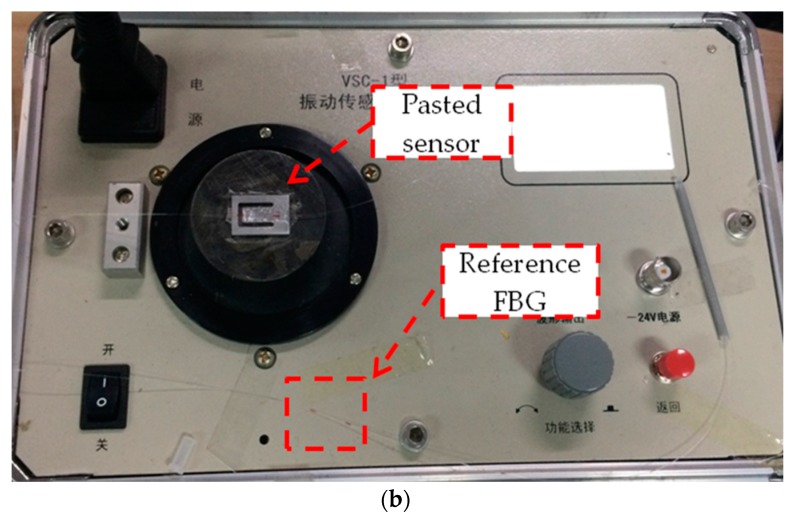
The equipments used in experiment. (**a**) The experimental setup. (**b**) The pasted sensor and reference FBG.

**Figure 20:**
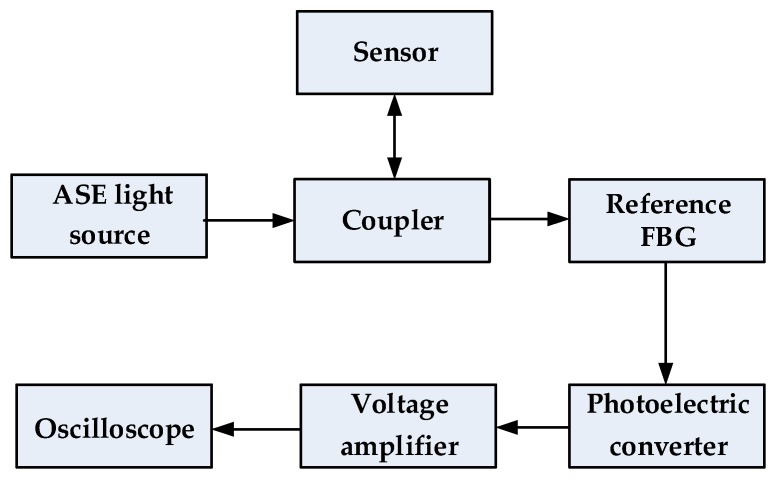
The demodulation method of the sensing system.

Using the processed sensor and light intensity demodulation method, some simple physical experiments have been done. Figure 19 shows the equipment and the sensor used in the experiments. Intensity demodulation method is used to demodulate the vibration signal. Broadband light is generated from the ASE light source and goes into the sensor through the coupler and reference FBG. The reflectance spectrum of the reference FBG is partly overlapping with that of the sensor. When the spectrum of the sensor changes with the vibration, the light intensity reflected by the sensor and the reference FBG will change accordingly [26], which can be used to demodulate the vibration or stress. The photoelectric converter transforms the light signals into electrical signals, which is enlarged by the 800-time voltage amplifier before being detected by the oscilloscope. The voltage amplifier is driven by the constant voltage source. The sensor is pasted on the vibrostand. The diagram of the demodulation method is shown in Figure 20.

**Figure 21:**
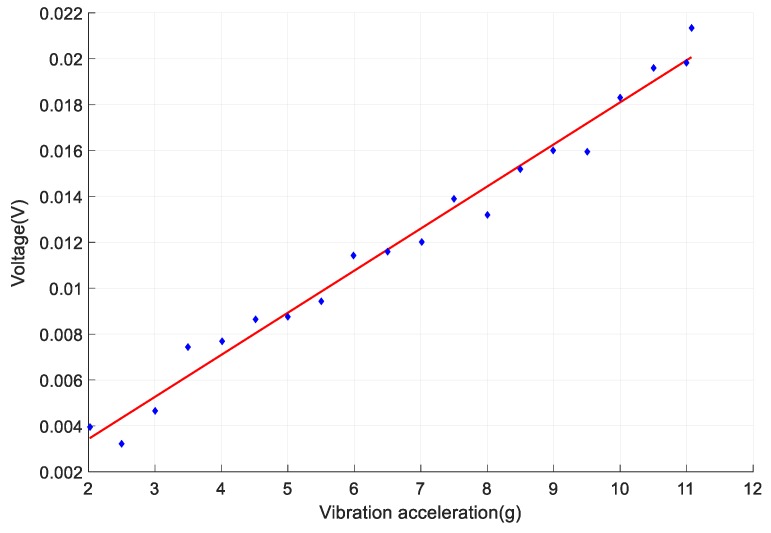
The output signal and the fitted line of vibration sensing.

Figure 21 shows the signal sensed by the sensor. The vibration frequency is set as 200 Hz. The blue points are the output voltage with different accelerations. The red line is a fitted line using least squares method. Its sensitivity is 18 mV/g. The vibration signal without the designed steel plate drowned in the noise of the demodulation circuit and could not be recognized. Thus, the sensing sensitivity of the designed sensor is much larger than that of normal FBGs.

#### 3.2.3. Sensitivity for Vibration Direction

**Figure 22:**
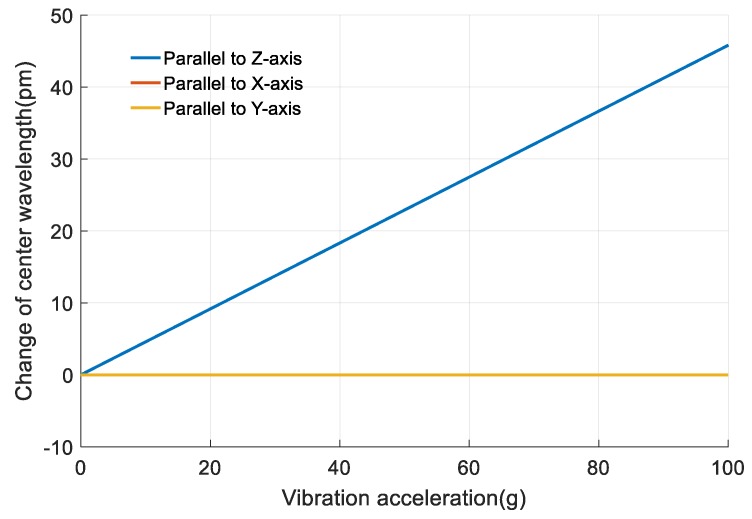
The output Δλ of the designed sensor with different vibration direction.

From the above analysis, the loaded vibration is parallel to the *Z*-axis. If the vibration is parallel to the *X*-axis or the *Y*-axis, the output *Δλ* of FBG 2 will be much smaller, as shown in Figure 22. *Δλ* caused by the vibrations parallel to *Z*-axis, *X*-axis and *Y*-axis are represented by *Δλ*_2*z*_, *Δλ*_2*x*_ and *Δλ*_2_*_δ_*, respectively. The curves of *Δλ*_2*x*_ and *Δλ*_2*y*_ coincide in Figure 22. When the vibration accelerations are the same, *Δλ*_2*x*_/*Δλ*_2*z*_ equals to 5.09 × 10^−10^ and *Δλ*_2*y*_/*Δλ*_2*z*_ equals 8.22 × 10^−12^, so vibrations parallel to the *X*-axis and *Y*-axis can hardly affect the output 5.09 × 10^−10^ *Δλ* of FBG 2 compared with vibrations parallel to *Z*-axis. The sensor is only sensitive to the vibration parallel to *Z*-axis. 

### 3.3. The Result of Finite Element Simulation

**Figure 23:**
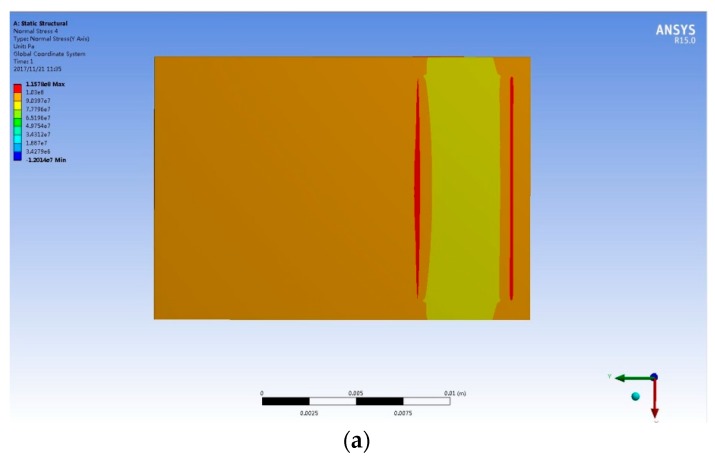

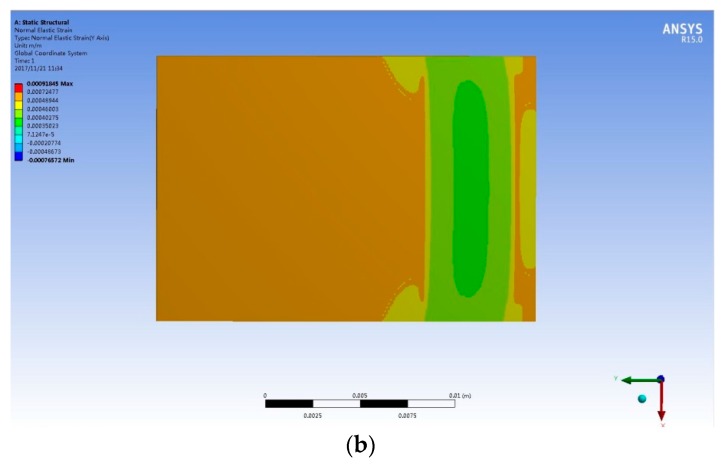
Results of the FEM for stress sensing. (**a**) The stress distribution of the measuring object; (**b**) The strain distribution of the measuring object.

**Figure 24:**
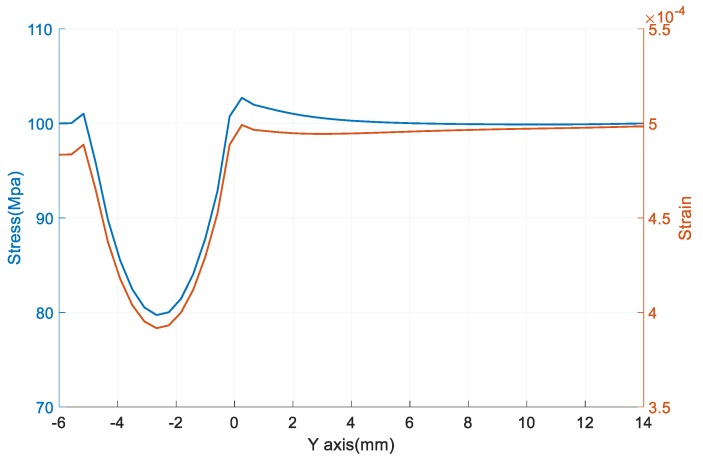
The stress and strain of the surface of the measuring object along the *Y*-axis.

FBG 1 is pasted on measuring object and it is under FBG 2. Its center coordinates are *y* = 2 mm and *x* = 0 mm. Figure 23 shows distribution of stress and strain along the *Y*-axis on the surface of the measuring object. Figure 24 shows the values of the strain and stress. It can be seen that the stress is close to the loaded pressure (100 Mpa) when the y-coordinate is larger than 2 mm. So the pasted sensor has little impact on the stress and strain distribution of the sensing area. 

### 3.4. The Sensor Performance for Measuring Stress

According to Section 3.3, the coordinates of the mid-point of FBG 1 are *y* = 2 mm and *x* = 0 mm. The strain at this point is taken as the average strain of FBG 1 [25]. To protect the designed sensor from over loading, its range for sensing stress is set as 0–100 Mpa.

#### 3.4.1. Measurement Linearity of the Sensor

**Figure 25:**
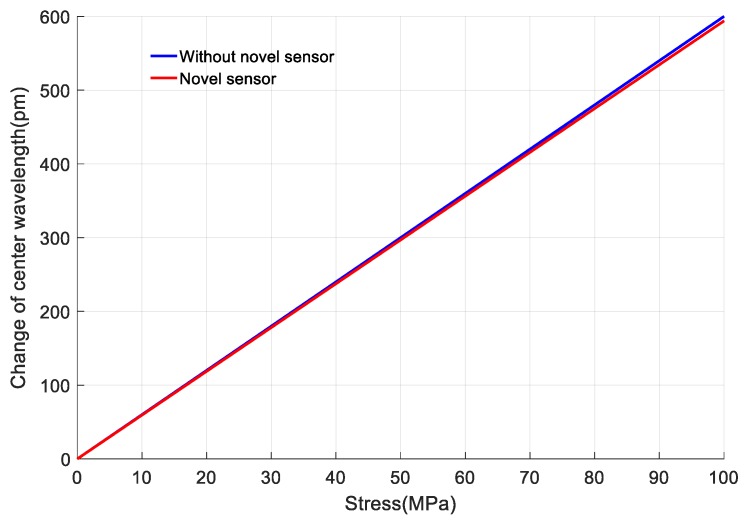
The relationship between the output Δλ of FBGs and loaded stress.

The paste sensor will slightly affect the surface stress distribution of the measuring object. According to Section 2.2 and Section 2.4, the relationship between Δλ of FBG 1 and the loaded stress is obtained. It is shown as the red curve in Figure 25. The blue curve represents Δλ of FBG 1 without using the designed stainless steel plate. The linearity error of the sensor is 1.02%. The designed sensor’s sensitivity for sensing stress is 5.94 pm/MPa.

#### 3.4.2. Sensitivity for Stress Direction

**Figure 26:**
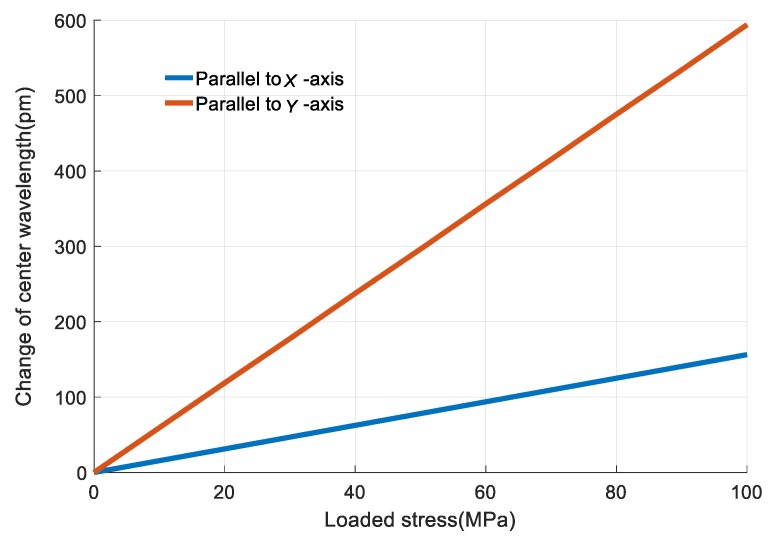
The output *Δλ* of the designed sensor with different stress direction.

In the above analysis, the loaded stress is parallel to *Y*-axis, as shown in Figure 6. If the stress is parallel to *X*-axis, the output *Δλ* of FBG 1 will be much smaller, as shown in Figure 26. *Δλ* caused by stress parallel to *Y*-axis and *X*-axis are represented by *Δλ*_1*y*_ and *Δλ*_1*x*_, respectively. With the same loaded stress, *Δλ_x_*/*Δλ_y_* = 0.26. It shows that the designed sensor is more sensitive to the stress parallel to *Y*-axis. So the *Y*-axis of the sensor is suggested to be used to sense the stress of measuring object. 

The sensor is designed to measure the object which is loaded with uniaxial stress in a known direction. The stresses of the measuring object in other directions are much smaller than that in the known direction, so they can hardly affect the sensing result while the sensor is sensing the uniaxial stress in the known direction. 

## 4. Conclusions

A sensor is proposed to measure vibration and stress at the same time, and improve the vibration sensing sensitivity significantly. The height of the sensor is 1 mm and the weight is about 1.15 g. It is helpful for sensors to be integrated, miniaturized and lightweight. Its working range is 0–6000 Hz for vibration sensing and 0–100 MPa for stress sensing. Its sensitivity are 0.46 pm/g for vibration sensing and 5.94 pm/MPa for stress sensing. Additionally, theoretical analysis of the sensor’s cantilever structure agrees with that of the FEM. The way the sensor pasted has little effect on the stress distribution of the cantilever structure. Compared with the general FBG which is pasted directly on the subject surface, the sensing sensitivity of the designed sensor for sensing vibration is increased by 26.2 times with a nonlinearity error of 0.77%. The sensor is only sensitive to the vibration parallel to *Z*-axis. The nonlinearity error of the sensor for stress sensing is 1.02%. 

In the following work, some further experiments need to be carried out to verify the theoretical analysis and FEM results. Above all, this work demonstrates that the designed sensor with two FBGs and a cantilever structure is a better choice for sensing vibration and stress simultaneously.

## Figures and Tables

**Table 1:** The geometry parameters and material properties parameters of the designed plate.

Geometry Parameters	Value	Material Properties	Value
*l* (m)	10 × 10^−3^	*E* (Pa)	1.93 × 10^11^
*b* (m)	5 × 10^−3^	ρ (kg∙m^−3^)	7750
*h* (m)	0.2 × 10^−3^	ν	0.31
*d* (m)	5 × 10^−3^		
*L* (m)	18 × 10^−3^		
*B* (m)	12 × 10^−3^		
*H* (m)	1 × 10^−3^		
